# The System Power Control Unit Based on the On-Chip Wireless Communication System

**DOI:** 10.1155/2013/939254

**Published:** 2013-05-30

**Authors:** Tiefeng Li, Caiwen Ma, WenHua Li

**Affiliations:** ^1^Xi'an Institute of Optics and Precision Mechanics of Chinese Academy of Sciences, Xi'an 710119, China; ^2^The Graduate University of Chinese Academy of Sciences, Beijing 100049, China

## Abstract

Currently, the on-chip wireless communication system (OWCS) includes 2nd-generation (2G), 3rd-generation (3G), and long-term evolution (LTE) communication subsystems. To improve the power consumption of OWCS, a typical architecture design of system power control unit (SPCU) is given in this paper, which can not only make a 2G, a 3G, and an LTE subsystems enter sleep mode, but it can also wake them up from sleep mode via the interrupt. During the sleep mode period, either the real-time sleep timer or the global system for mobile (GSM) communication sleep timer can be used individually to arouse the corresponding subsystem. Compared to previous sole voltage supplies on the OWCS, a 2G, a 3G, or an LTE subsystem can be independently configured with three different voltages and frequencies in normal work mode. In the meantime, the voltage supply monitor, which is an important part in the SPCU, can significantly guard the voltage of OWCS in real time. Finally, the SPCU may implement dynamic voltage and frequency scaling (DVFS) for a 2G, a 3G, or an LTE subsystem, which is automatically accomplished by the hardware.

## 1. Introduction

With the rapid increase of complexity and size of OWCS, the power consumption issue for the OWCS is increasingly becoming critical and needs to be solved quickly. As we know, the duration and stability of voltage supply are two factors that have an important impact on the power performance of the OWCS. The duration and stability of voltage supply are two factors that have an important impact on the power performance of the OWCS. In the first instance, the duration period depends on overall power consumption of the whole OWCS when the total amount of the battery's power is fixed and unchanged. An excess of power consumption can accelerate the aging of the OWCS and cut down the battery life. On the other hand, the power density of OWCS is continuously increasing with the scaling of each technology generation. It is necessary for the whole OWCS to reduce the dissipated heat because high heat dissipation increases instability, which can lead to the drastic fluctuation of voltage supply and the crash of OWCS in a short time. Early in the OWCS design stage when there is a lack of conception of saving energy, it is usually ignored by designers because the negative influence that is directly derived from power consumption is very small. However, in the development of wireless communication and the integrated circuit technology, the design of OWCS is more complicated and consumes more energy. In this case, it is often desirable to minimize power consumption to maximize OWCS lifetime. In this paper, we intend to deal with the power consumption issue of the OWCS with an efficient hardware approach and see how it is different from others' designs. In doing so, we put forward one new SPCU concept that is based on the OWCS [1, 2], which can work in both the active and sleep mode according to the actual requirement. The differences between the novel SPCU and the old design are emphasized in the introduction. Firstly, in the traditional design, software scheduler method in the operating system is still popular in reducing power consumption, which estimates CPU workload according to the frequencies of calling scheduler [3–9]. The main reason that most engineers adopt software scheduler is that they are not real CPU designers and are unable to change CPU hardware architecture at all. Thus, they have to lower power consumption in the operating system via software compensation method. However, with the quick development of CPU technology, the dominant frequency of current CPU has achieved more than 1.5G. For example, the dormant frequency of ARM Cortex A9 is about 1.6G. Obviously, this software approach is not enough in dynamic environments because it cannot accurately trace the status of CPU workload in the deadline when the frequency of CPU is higher than before. By contrast, the new SPCU that is fully presented from hardware side has a remarkable improvement in saving power. Because SPCU is integrated into the OWCS with three different voltages and frequencies, the various performance requirement of CPU can be rapidly responded by SPCU at the first moment; thus, the speed and accuracy of tracking CPU workload are largely improved in essence. The most significant is that all tasks of tracking CPU workload and predicting CPU performance can be finished by hardware automatically, which can transparently lessen the software burden of operating system in the OWCS. Secondly, many new features are firstly used in SPCU. For instance, the GSM sleep timer, the hardware DVFS, the supply voltage monitor, the reset status recorder, and the aging monitor. Thirdly, some features are optimized, such as the clock controller. Those features will be depicted in detail below.

Generally speaking, the contribution in this paper is that new SPCU aims at novelty in the realization of hardware architecture, instead of following the beaten path of conventionality. In actual application, it is proved that the reliable SPCU can keep the OWCS work with lower power consumption and increase the OWCS working cycle better. In our design, the SPCU mainly includes the transition state machine of power mode, the clock controller, the sleep timer, the voltage supply monitor, the reset and wake-up circuit, the voltage converter, the hardware DVFS, and the aging monitor.

## 2. The State Machine of Power Mode Transition 

In conventional design [10], the state machine of power mode transition has two drawbacks: (1) only one sleep mode is deep mode, (2) the subpower domains are not switched off during deep sleep. For the previous OWCS, shallow sleep is critical for diminishing power. In addition to the shallow sleep requirement, the subpower domains should be switched off when voltage of CPU is automatically switched off during deep sleep. From the point of view of saving power consumption, it is necessary to minimize power consumption during deep sleep. 

The new state machine of power mode transition with improved sleep mode and subpower domains is applied in the SPCU, which has two key advantages: (1) there are two power modes in the OWCS, which are the active mode and the sleep mode consisting of shallow and deep sleep mode, (2) it is recursive between subpower domains and their corresponding CPU power domain. In other words, all subpower domains in the SPCU will be compulsorily switched off if CPU power supply is switched off during deep sleep. Here, we just take the 2G subsystem as an example because the power mode transition of the 3G and the LTE are similar to the 2G's power transition.

**Figure 1:**
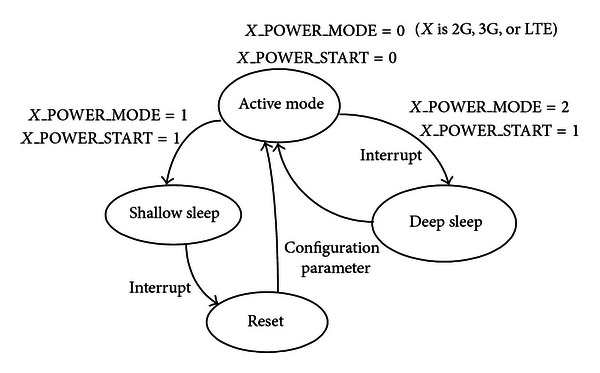
The state machine of power mode transition.

In Figure 1, with the beginning of power on, the whole OWCS normally works with active mode. At this moment, the power mode of the 2G subsystem can be indicated by the signal 2G_POWER_MODE because the value of signal 2G _POWER_MODE is zero during the active mode. In this case, depending on our needs, the voltage supply of 2G CPU may be initialized with the three-voltage option (*V*_High_ = 1.25 V, *V*_Medium_ = 1.0 V, *V*_Low_ = 0.80 V). In default, the voltage of 2G CPU is supplied with *V*_Medium_ and the clock of 2G CPU is provided with 360 MHz that is consistent with the medium voltage. As soon as the signal 2G_POWER_MODE is set to one and the signal 2G_POWER_START is set to one, the 2G subsystem will immediately enter the shallow sleep mode, in which the voltage supply of 2G CPU will not be switched off but the clock of the 2G CPU is decreased from 360 MHz to 32.768 kHz that is generated by on-chip oscillator [11]. As a result, the whole 2G subsystem reduces power consumption by slowing the clock frequency. Furthermore, the 2G subsystem will be woken up from the shallow sleep and be restored to the previous state if the interrupt happens. After the 2G subsystem is woken up, the clock frequency of 2G CPU will go back to the normal frequency. On the other hand, when the signal POWER_MODE is set to two and the signal 2G_POWER_START is set to one, the 2G subsystem will enter the deep sleep from active mode, see also Figure 1.

Especially, the power and clock of the 2G CPU is fully switched off during the deep sleep period, and its corresponding sub-power domains are also switched off. This makes the data contents of memory lost in deep sleep mode. In order to avoid losing data, the current data should be saved into the memory by setting retention mode before the deep sleep is requested. However, the power of the on-chip oscillator never be switched off in both sleep modes because it provides 32.768 kHz clock to awake circuit. The 2G subsystem will be woken up from the deep sleep and be rebooted as soon as the internal or external interrupt happened. After the 2G subsystem is rebooted, its clock frequency is back to the normal 360 MHz. 

## 3. The Clock Controller

**Figure 2:**
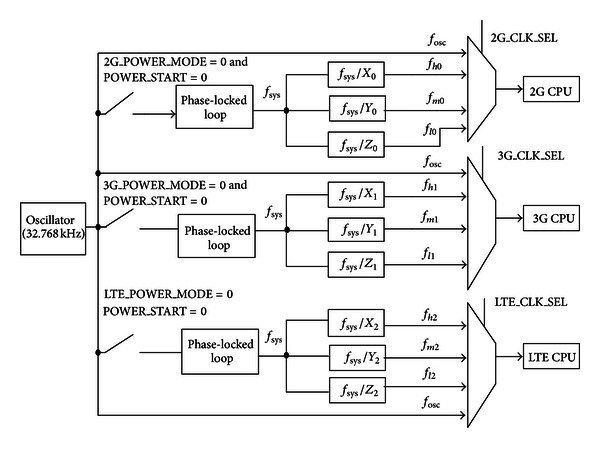
The clock controller.

 As shown in Figure 2, the clock controller includes three phase-locked loops (PLLs) [11, 12], three clock dividers, and three digital multiplexes. In the previous clock controller, a Phase-Locked Loop (PLL) is used for 2G and 3G subsystem [13]. However, when LTE subsystem is merged into the smart OWCS, power saving has to be considered again [14, 15]. Here, we present an optimized clock controller that has three independent PLLs for DVFS. Compared with conventional design, this gives us much more flexibility to execute DVFS with three frequencies and voltages because every PLL is dedicated to 2G, 3G, or LTE subsystem. Moreover, every PLL can convert a low-frequency external clock signal that is generated by the on-chip 32.768 kHz oscillator to a high-speed internal clock for maximum.

Depending on the different frequency requirement, the clock frequency output may be configured by programming desired *N*, *P*, and *K* values according to (1). Normally, only if the signal POWER_MODE and the signal POWER_START in every subsystem (2G, 3G or LTE) are set to 00B at the same time, the on-chip 32.768 kHz oscillator will start to output clock to the corresponding PLL. For example, if 2G_POWER_MODE and 2G_POWER_START are set to 00B, the 32.768 kHz oscillator will output clock to the PLL of 2G subsystem. However, if the subsystem (2G, 3G or LTE) is in the deep sleep mode, the on-chip 32.768 kHz oscillator is still working but it stops providing the clock to the corresponding PLL so that no clock signal is coupled to the subsystem's CPU. Here, the generalized PLL output in three subsystems can be defined as

(1) $$\begin{array}{c} f_{\text{sys}}=f_{\text{osc}}*\frac{N}{P*K}, \end{array}$$

where *N*, *P*, and *K* are integers predefined according to the actual requirement. The CPU clock is derived from the oscillator clock (*f*_osc_), multiplied by *N*, divided by *P*, and divided by *K*. The clock output from the 2G clock dividers can be stated as follows:

(2) $$\begin{array}{c} f_{h0}=\frac{f_{\text{sys}}}{X_{0}},\\ f_{m0}=\frac{f_{\text{sys}}}{Y_{0}},\\ f_{l0}=\frac{f_{\text{sys}}}{Z_{0}}, \end{array}$$

where *X*_0_, *Y*_0_, and *Z*_0_ are the integers and *X*_0_ < *Y*_0_ < *Z*_0_, *f*_*h*0_ represents the high input clock frequency of the 2G CPU, *f*_*m*0_ shows the medium input clock frequency of 2G CPU, *f*_*l*0_ means the low-input clock frequency of the 2G CPU. Moreover, we can conclude according to (2) that *f*_*h*0_ > *f*_*m*0_ > *f*_*l*0_.

The clock output from the 3G clock dividers can be written as follows:

(3) $$\begin{array}{c} f_{h1}=\frac{f_{\text{sys}}}{X_{1}},\\ f_{m1}=\frac{f_{\text{sys}}}{Y_{1}},\\ f_{l1}=\frac{f_{\text{sys}}}{Z_{1}}, \end{array}$$

where *X*_1_, *Y*_1_, and *Z*_1_ are the positive integers and *X*_1_ < *Y*_1_ < *Z*_1_, *f*_*h*1_ represents the high input clock frequency of the 3G CPU, *f*_*m*1_ shows the medium input clock frequency of the 3G CPU, and *f*_*l*1_ stand for the low-input clock frequency of the 3G CPU. Further, we can also derive from the above Equations (3) that *f*_*h*1_ > *f*_*m*1_ > *f*_*l*1_.

The clock output from the LTE clock dividers can be defined as follows:

(4) $$\begin{array}{c} f_{h2}=\frac{f_{\text{sys}}}{X_{2}},\\ f_{m2}=\frac{f_{\text{sys}}}{Y_{2}},\\ f_{l2}=\frac{f_{\text{sys}}}{Z_{2}}, \end{array}$$

where *X*_2_, *Y*_2_, and *Z*_2_ are the positive integers and *X*_2_ < *Y*_2_ < *Z*_2_, *f*_*h*2_ represents the high input clock frequency of the LTE CPU, *f*_*m*2_ shows the medium input clock frequency of the LTE CPU, and *f*_*l*2_ means the low-input clock frequency of the LTE CPU. Beyond this, we can similarly deduce on the basis of (4) that *f*_*h*2_ > *f*_*m*2_ > *f*_*l*2_.

For the three digital multiplexes, the control signals (2G_CLK_SEL, 3G_CLK_SEL and LTE_CLK_SEL) can be set with three different frequency clock (*f*_high_, *f*_Medium_, and *f*_Low_) that are absolutely synchronous to three voltage configurations (*V*_High_, *V*_Medium_, and *V*_Low_); that is, when one kind of voltage supply is configured on the subsystem of the OWCS, the voltage configuration also can be passed to the signal *X*_CLK_SEL in the meantime. Also, during the shallow sleep, the clock controller works with bypassed mode, where the clock fosc can be directly bypassed to the digital multiplexes without going through the PLLs and can be chosen as the clock output of the multiplex by setting the control signal *X*_VSET to 11B.

The clock of the 2G, the 3G, or the LTE can be selected by the digital multiplexer. The formula of the clock output can be described as follows:

(5) $$\begin{array}{c} f_{\text{out}}=\{\begin{array}{cc} {f_{l}}_{n}\; & \text{if}\;\;X\_\text{CLK}\_\text{SEL}=00\text{B}\\ f_{mn} & \text{if}\;\;X\_\text{CLK}\_\text{SEL}=01\text{B}\\ f_{hn} & \text{if}\;\;X\_\text{CLK}\_\text{SEL}=10\text{B}\\ f_{\text{osc}} & \text{others}, \end{array} \end{array}$$

where *X* is 2G, 3G, or LTE, *n* is 0, 1, or 2. 

## 4. The Sleep Timer 

### 4.1. The Real-Time Sleep Timer

In the conventional design, sleep time is mainly controlled by external timer [15–17]. If external timer is occupied by other module during sleep, the sleep time will not be controlled again. In order to resolve this problem, the real-time sleep timer is newly introduced in our SPCU. 

**Figure 3:**
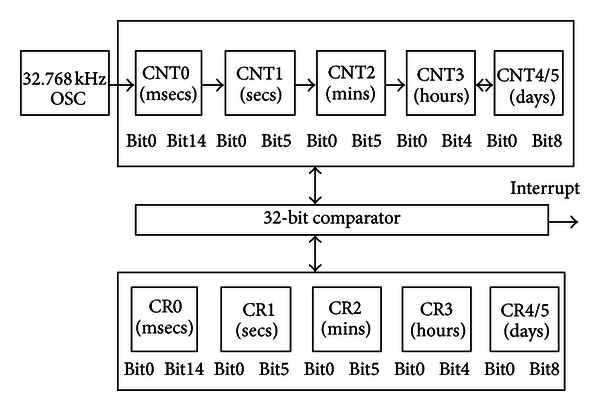
The real-time sleep timer block.

The main concept is that the 2G, the 3G, or the LTE subsystem has its own real-time sleep timer that can precisely control the sleep time, which gives us great flexibility when we control the sleep time of the 2G, the 3G, or the LTE subsystem. With reference to Figure 3, the real time sleep timer includes the milliseconds counter, the seconds counter, the minutes counter, the hours counters and the days counters. In default, the milliseconds counter CNT0 are 15 bits, which are set to an initial value of 000_0000_0000_0000B. One increment of the timer is then made for every cycle of the input clock. With an input clock frequency of 32.768 kHz which is from on-chip oscillator, one second will be equivalent to an overflow of a 15-bit timer. Under this condition, the register CNT5-CNT0 actually holds the real-time value in the range of milliseconds, seconds, minutes, hours, and days. Register CNT0 has 15 bits that hold the range of milliseconds of the clock. The register CNT2 and CNT1 are 6 bits, which hold the minutes and seconds of the clock. The seconds and minutes counter will be reset to zero after 60 counts. The CNT3 register holds the hours of the clock. It resets to zero after 24 counts. The registers CNT4 and CNT5 hold the days for the clock. They count for one year before it is reset to zero. One year could be 365 or 366 days. Bit TYR is used to select between a normal year and a leap year.

The real time sleep timer has to be initialized before the sleep mode start. While it is in operation, the contents registers (CR0-CR5) register will be compared to the real time sleep timer counter (CNT0-CNT5) that is programmable with any values that indicates the seconds, minutes, hours and days. An interrupt will be generated in sleep mode when the contents are equal, which will rouse the 2G, the 3G, or the LTE subsystem from the sleep mode. 

### 4.2. The GSM Sleep Timer

**Figure 4:**
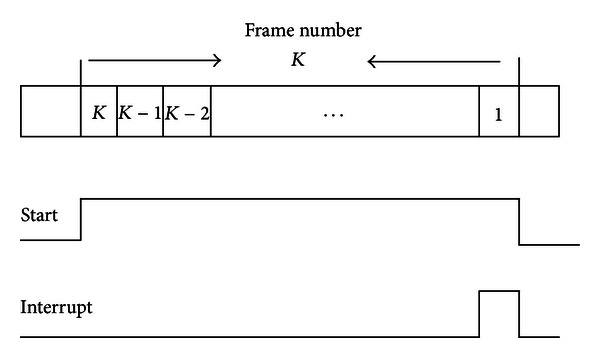
The GSM sleep timer.

The resynchronization problem of GSM system always arises in previous 2G subsystem [18], where there is time mismatch between GSM system and base station when the OWCS is woke up from sleep mode; the main reason is that GSM system [14, 15] has been reset since whole OWCS woke up. As a result, the parameter of GSM system has to be set again in order to synchronize with the base station. To cope with this shortcoming in the OWCS, the GSM sleep timer is firstly introduced in the SPCU, which is one special sleep timer that only belongs to the 2G subsystem. It is emphasized that the GSM sleep timer is basically independent from the system sleep mode, so that GSM sleep timer can be dedicated to work as a wake-up source. The time unit of the GSM sleep timer is based on the time division multiple access (TDMA) frame. Every TDMA frame time is about 4.615 milliseconds that are synchronized with the GSM communication network [16–18]. The advantage of the GSM sleep timer is that it offers a convenient hardware solution to switch off the 2G subsystem for an accurate integer multiple of a TDMA frame (sleep duration). During the sleep mode, the GSM sleep timer controller is clocked with 32.768 kHz that is from on-chip oscillator. The TDMA frame number is predefined via software before the 2G subsystem enters the sleep mode. When the control signal START is set to the high level, the GSM sleep timer controller will immediately start to run, which will decrease one with every 4.615 milliseconds until the TDMA frame number is zero. In the meantime, the control signal START will become low and the SPCU will send an interrupt to the 2G CPU so that the 2G subsystem will be subsequently woken up.  Figure 4 explains the exact timing of the whole GSM sleep timer between the active and the sleep mode. In the single antenna interference cancellation (SAIC) mode, the sleep duration accuracy was improved from 1.04 to 0.52 ppm compared to earlier implementations.

## 5. The Wake-Up and Reset

 As we know, there is no LTE subsystem in the earlier OWCS [19, 20], so the old SPCU design does not cover the power system of LTE subsystem. The improvement in the new SPCU is that the LTE subsystem can be taken as an independent wake-up source, which not only can wake up the 2G or 3G subsystem but it also can be woken up from sleep by 2G or 3G subsystem. Furthermore, soft reset is specially introduced in order that subsystem can go back to the previous running state from shallow sleep. Unlike soft reset, hardware reset is dedicated to the OWCS system crash. Moreover, reset behaviour cannot be recorded by SPCU in the conventional design, so it is not convenient to check wake-up source and reset type. The advantage in new SPCU is that reset status recorder and wake-up status recorder are merged and implemented well in our design.

### 5.1. The Wake-Up Source

**Figure 5:**
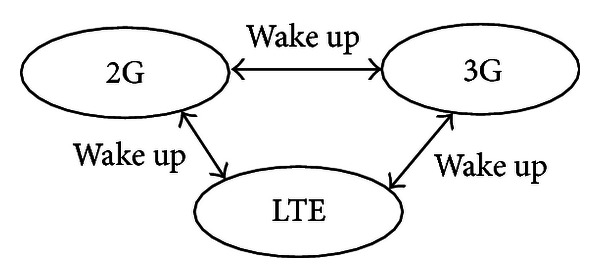
The 2G, the 3G, and the LTE wake up relationship.

For the 2G, the 3G, or the LTE subsystem, its wake-up source is composed of internal and external source. The internal sources are the three independent sleep timers and the DSP. The external sources are the chip pins, the 3G, and the LTE. For the 3G and the LTE, they also can be woken up by the 2G when they are in sleep mode state. The flexible wake-up relationship among the three subsystems is given in Figure 5.

 After the 2G, the 3G, or the LTE subsystem is woken up and is reset, the SPCU will not be reset yet, and then the wake-up status register of the SPCU can record which source is triggered. The wake-up status register has to be cleaned before going into next sleep mode.

### 5.2. The Reset Type

#### 5.2.1. The Soft Reset

When the 2G, the 3G, or the LTE subsystem separately enters the shallow sleep mode, it can be woken up and be restored to the previous state via soft reset. Note that the soft reset is not effective to the deep sleep.

#### 5.2.2. The Hardware Reset

The hardware reset is a special reset in the OWCS because it can deal with the urgent case. After the 2G, the 3G, or the LTE subsystem enters the deep sleep from active mode, the whole on-chip system will be woken up and rebooted if reset key is pressed. This kind of hardware reset is not frequently used in the actual application because it resets the 2G, the 3G, and the LTE subsystem at the same time.

#### 5.2.3. The Wake-Up Reset

Compared to the soft reset, the wake-up reset plays an important role in the on-chip wireless communication chip, which is often used in both the shallow sleep and the deep sleep. When the 2G, the 3G, or the LTE subsystem is woken up from the shallow sleep or the deep sleep by the interrupts, the reset status register will record reset status and the wake-up status register will record which source is working.

## 6. The Voltage Supply Monitor

In traditional OWCS design, the system voltage supply is not monitored exactly in real time [21–23]. From the point of view of power supply security, SPCU must be sensitive to the changing voltage supply so that the OWCS system can get timely prewarning to eliminate some potential risks. In this context, the distinctive supply voltage monitor is firstly introduced in this paper in order to enhance guarding on system voltage. 

**Figure 6:**
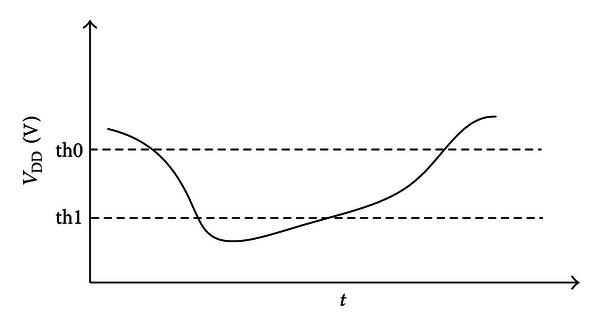
The voltage supply monitor.

In our SPCU, the basic functionality of the supply voltage monitor is primarily targeted as a measurement and analysis tool during normal operation of the chip. It gives a permit to measure and guard the internal subsystem CPU supply voltage on the chip for every individual CPU clock cycle. Furthermore, it is exposed to the worst operating condition that the highest on-chip voltage drops. Meanwhile, it is optionally fed up with three subsystem CPU clock frequency. The major benefit of the supply voltage monitor is that it can ensure that each subsystem CPU is supplied with the normal voltage in real time. Once the 2G, the 3G, or the LTE CPU voltage is less than a fixed threshold value in the active mode, the voltage supply monitor will send one interrupt signal to the subsystem CPU and configure the voltage again (see Figure 6). The voltage output from the voltage converter is adjusted in such a way that the supply voltage drops below the critical voltage threshold even under worst-case conditions. The supply voltage monitor has two threshold voltages, which means the supply voltage of subsystem (2G, 3G, or LTE) has double protection. For the two threshold voltages, they can be configured in advance by means of setting the voltage control register. The predefined threshold voltage formula can be stated as follows:

(6) $$\begin{array}{c} \text{Threshold}=0.80+0.01*X, \end{array}$$

where *X* is from 0 to 40. The precision of two threshold voltages can be guaranteed in real operation according to (6).

## 7. The Voltage Converter

The voltage converter plays an important role in supplying voltage for the OWCS. In traditional design, a voltage converter is used to provide voltage supply to 2G or 3G subsystem [23–26]. However, in practice, it sometimes leads to voltage requirement conflict because 2G and 3G subsystems work in parallel.

Unlike common voltage converter, new voltage converter has the three independent step-down converters, which are the 2G voltage converter, the 3G voltage converter, and the LTE voltage converter. Particularly, the three step-down converters can operate in two modes. One mode is the pulse frequency modulation (PFM) mode with a lower quiescent current at limited output current, the other mode is the pulse width modulation (PWM) mode with full output current but a higher quiescent current. Throughout the sleep phases, the PFM mode is required so that the current consumption of the on-chip system and the memory is very low (≪1 mA). As a result, the power consumption is minimized. Whenever the subsystem is woken up, the mode of the step-down converter is forced to switch to PWM mode.

**Figure 7:**
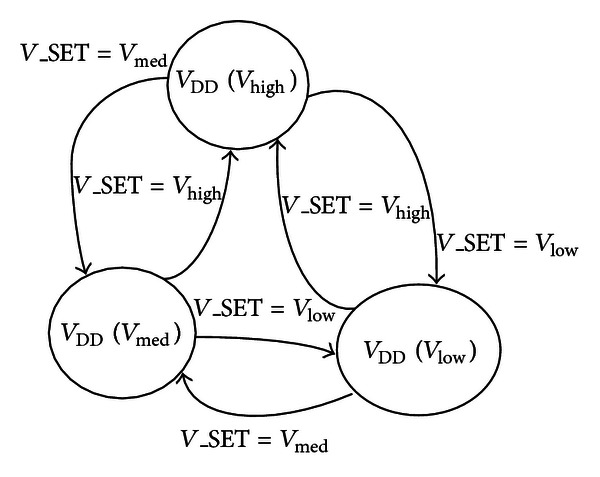
The voltage transition.

**Figure 8:**
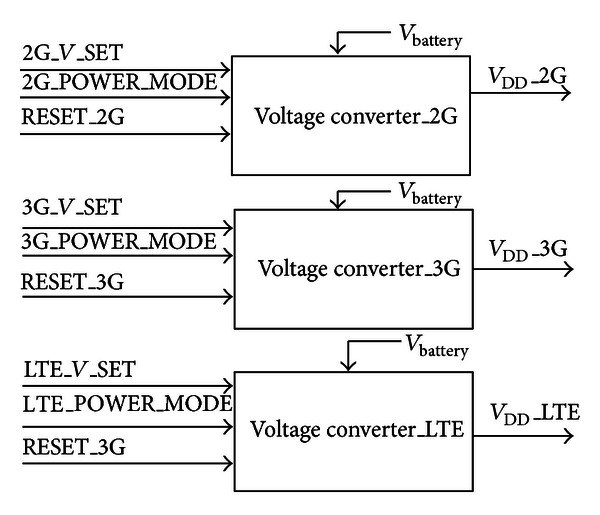
The voltage converter block.

It is shown in Figure 7 that the voltage converter output of every subsystem (2G, 3G, LTE) is configured with three voltages (*V*_Low_, *V*_High_, and *V*_Medium_) according to the input signal *V*_SET. Three voltage configuration details by the 2-bit width signal *V*_SET is shown in Table 1. Furthermore, the voltage converter output will be switched off in deep sleep mode. It will be powered on again when the input signal RESET_*X* is active by waking up or hardware reset. Hence, the voltage converter can reduce the power consumption of the on-chip system with flexible voltage configuration. Figure 8 presents the three voltage transitions for three subsystems CPU voltage.

## 8. The DVFS Theory

Currently, the researchers and designers are still adopting software method predicts performance requirement of CPU according to the sequence of event priority in the software scheduler [3–9]. Although it can low power in a way, it is only efficient on the case that CPU frequency is not high, it will become difficult with the increment of CPU frequency because the software method cannot correctly respond to the high frequency of CPU, so it is also failed to estimate the performance requirement of CPU in time [27, 28]. To overcome this, the hardware DVFS is fully introduced in this paper, which has very fast tracking and response speed on CPU behaviour. 

For the OWCS, its dynamic power formula is stated as follows:

(7) $$\begin{array}{c} P=\alpha CV^{2}f, \end{array}$$

where *α* represents the percentage of logic cell between 0 and 1 switching, *C* is a constant that represents the circuit load, *V* represents the CPU voltage, and *f* represents the CPU frequency. 

**Figure 9:**
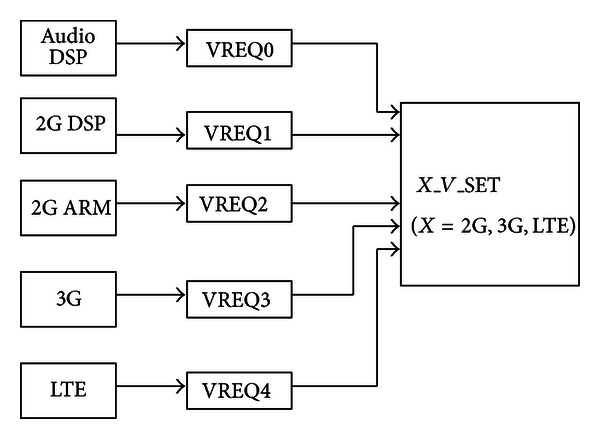
The DVFS structure.

We can easily know according to (7) that the power of CPU can be lowered by reducing the voltage and frequency. To some degree, it is tedious to change the dynamic voltage and frequency by means of software. In order to cut down the software overloads on the DVFS, the 2G, the 3G, the LTE, and the DSP subsystems can request the DVFS that is finished by the hardware in parallel or respectively. The structure of hardware DVFS in the active mode is given in Figure 9. With reference to Figure 9, the function of DVFS can be enabled by setting the signal VREQ*n* (*n* is 0, 1, 2, 3) that is 1-bit width. Once the signal VREQ*n* is set, the voltage of the subsystem CPU or DSP need not be adjusted by the software again and again. In comparison to the previous software design, it firstly strengthens the accuracy of voltage and frequency estimation. Secondly, it lightens the load of CPU timing tracking in a way. 

**Figure 10:**
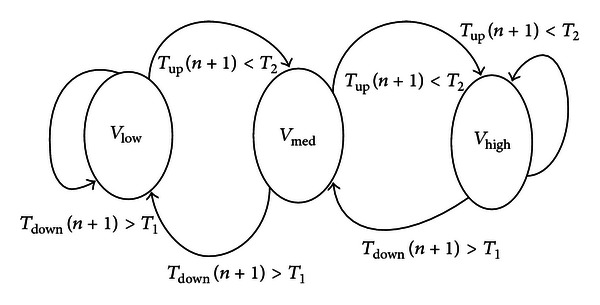
The intelligent transition for DVFS.

In the progress of the DVFS, the SPCU can accurately predict the voltage that is needed in the next period of time according to the current CPU idle time, which supports two scaling steps (down, up). Each CPU of the three subsystems can be separately configured with the scaling down threshold value (*T*1) and the scaling up threshold value (*T*2). It is worth emphasizing that the two threshold values of the CPU idle time have to be set before the DVFS is requested. In the meantime, the moving average algorithm (MAA) is firstly adopted in the DVFS. The MAA not only tracks and samples the idle time of the every CPU with small enough intervals but also executes the accumulation and average calculation of the idle times. The MAA formula is given as follows:

(8) $$\begin{array}{c} T_{\text{up}}\left(n+1\right)=\frac{1}{N}\sum_{k=0}^{N-1}T\left(n-k\right),\\ T_{\text{down}}\left(n+1\right)=\frac{1}{M}\sum_{k=0}^{M-1}T\left(n-k\right), \end{array}$$

where *n*, *M*, and *N* are the positive integers and usually *M* > *N* > 0. *T*_up_(*n* + 1) stands for the average of the idle time from the sampling timing 0 to *N* − 1, *T*_down_(*n* + 1) stands for the average of the idle time from the sampling timing 0 to *M* − 1. Based on (8), the voltage and clock of CPU scaling down step condition is fulfilled if *T*_down_(*n* + 1) > *T*1. Similarly, the voltage and clock of CPU scaling up step condition is fulfilled if *T*_up_(*n* + 1) < *T*2.  Figure 10 shows the specific automatic transition for the DVFS. 

The DVFS has its own timer that can be set to the expected maximum voltage settling time. For example, if a voltage ramping slew rate of 5 mV per microsecond is used in changing to the adjacent voltage, it only takes 40 microseconds to stabilize from *V*_Low_ (0.8 V) to *V*_Medium_ (1.0 V). Whenever the voltage scaling timer elapses, an interrupt can be triggered. 

As shown in Table 2, it is obvious that the power consumption of each CPU is reduced with the DVFS in the actual test. Furthermore, compared with conventional software way (CSW) [29–31], the hardware DVFS has the absolute advantage in saving energy. Thus, we can clearly get a conclusion that the hardware DVFS is an efficient and smart way to save power. In Future, the hardware DVFS will be dominant in the OWCS because of the high efficiency. 

## 9. The Aging Monitor

It is meaningful for designers to analyze the important aging data so as to optimize the power system of OWCS. But in the conventional OWCS, the aging monitor has never been used successfully because of its implement complexity [31]. To solve this problem, a novel aging monitor is exactly descripted in this part.

As the OWCS ages, the reliability of internal components begins to diminish. The OWCS ages in operational use during which the internal components are exposed to varying operational temperature and voltages. In fact, the effects of aging are proportional to the cumulative temperature and voltages experienced during use. So internal components which operate at higher temperatures and voltages age faster and deteriorate quicker than those components experiencing more moderate temperatures and voltages. To a certain degree, it is challenging to monitor the age of target circuit component effectively. To solve it in time, we give the particular aging monitor concept to the OWCS.

**Figure 11:**
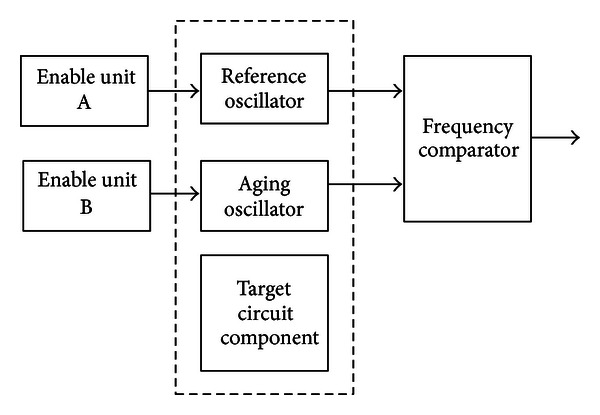
The aging monitor block.

In Figure 11, the age of the target circuit component in the OWCS may be monitored by using at least one aging monitor that includes one reference oscillator circuit and one aging oscillator circuit. For the aging oscillator circuit, it comprises a ring oscillator that generates aging clock signal having an aging frequency *f*_AGE_ that may change over time. Enable unit A is coupled to selectively enable or disable aging oscillator circuit. The aging oscillator circuit only generates aging clock signal when enabled. One or more components in the aging oscillator circuit may degrade over time when stressed. The degradation of those components may cause the aging frequency *f*_AGE_ to change. The aging oscillator circuit is positioned to proximate to the target circuit component. such that oscillator circuit and target circuit may experience the similar operational stress (e.g., temperature, voltage, etc.). In a word, the aging oscillator circuit and the target circuit component are exposed to an identical operating environment. Thus, degradation in the aging oscillator circuit and the target circuit component will be correlated.

In the same way, the reference oscillator has a ring oscillator that generates reference clock signal having a reference frequency *f*_REF_ that may change over time. Enable unit B is coupled to selectively enable or disable reference oscillator circuit. For the reference oscillator circuit, it can be enabled for short periods of time, just long enough to compare *f*_REF_ of reference clock signal with *f*_AGE_ of aging clock signal. When reference oscillator circuit is disabled, the components in the reference oscillator circuit are not stressed and are electrically isolated from the target circuit component. Thus, when the reference oscillator circuit is disabled, it does not experience the aging effects experienced by the target circuit component. Related to the cumulative operating time of the target circuit component and the aging oscillator circuit, the reference oscillator circuit is operated for very short periods of time so that it is not stressed significantly.

In Figure 11, the frequency comparator is coupled to receive and compare reference clock signal and aging clock signal. In response, the frequency comparator generates an age signal that is proportional to the operation age of target circuit component. Age signal generated by the frequency comparator may then be analyzed. The OWCS has multiple aging monitors that track operational age of the multiple circuit components, such as memory controller, subsystem CPU. Typically, the subsystem CPU may be specially measured by multiple aging monitors.

**Figure 12:**
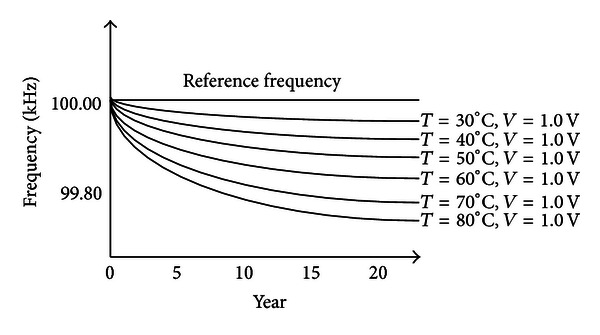
The aging progress.

According to Figure 12, we clearly explain that the aging effect is closely related to the operational temperature, voltage supply, and time. Assume that the environment temperature and voltage supply is not unchanged; as time goes, the aging effect becomes more apparent than before. In summary, the aging test in the OWCS is an indispensible flow that still needs to be improved in the future. 

## 10. Conclusion

With the reliability and the flexibility of the SPCU, each subsystem of the OWCS can be easily provided with the three different voltage and clock and request the DVFS. Besides, the sleep time can be precisely performed by two kinds of the sleep timers during the sleep mode; the voltage supply of each subsystem in the OWCS can be monitored in real time. It is worth noting that the new design can be used to solve the problem on how to save the power consumption and how to overcome instability of voltage supply. In general, it is obvious that the SPCU can make the OWCS save the power and monitor the age of OWCS well, which has been successfully used in the OWCS. Considerable more work, hopefully, will be done in this area on how to achieve the lowest power consumption in the OWCS by this method provided in this paper.

## Figures and Tables

**Table 1:** The binary voltage configuration.

Voltage configuration type	Binary value
*V*_low_	00B
*V*_med_	01B
*V*_high_	10B

**Table 2:** Saving power with the DVFS.

CPU access to memory	DVFS and CSW are disabled	CSW is enabled	Saving power	DVFS is enabled	Saving power
2G CPU	280 mW	265 mW	5.3%	221 mW	21.1%
3G CPU	198 mW	176 mW	11.1%	157 mW	20.7%
LTE CPU	183 mW	168 mW	8.2%	141 mW	23%
